# FKBP5 and NR3C1 epigenetic signatures are related to HPA-axis function in children and adolescents exposed to childhood maltreatment

**DOI:** 10.1192/j.eurpsy.2026.10242

**Published:** 2026-07-31

**Authors:** N. San Martín-González, D. Czamara, M. Acosta-Díez, L. Marqués-Feixa, H. Palma-Gudiel, S. Romero, M. Rapado-Castro, I. Zorrilla, H. Blasco-Fontecilla, B. Arias, S. Papiol, L. Fañanás

**Affiliations:** 1 Department of Evolutionary Biology, Ecology and Environmental Sciences, University of Barcelona, Barcelona; 2 Centre for Biomedical Research Network on Mental Health (CIBERSAM), Madrid; 3 Biomedicine Institute of the University of Barcelona (IBUB), Barcelona, Spain; 4 Department Genes and Environment, Max Planck Institute of Psychiatry, Munich, Germany; 5 Barcelonaßeta Brain Research Center (BBRC); 6 Department of Child and Adolescent Psychiatry and Psychology, Institute of Neuroscience, Hospital Clínic de Barcelona; 7 Institut d’Investigacions Biomediques August Pi i Sunyer (IDIBAPS), Barcelona; 8 Department of Child and Adolescent Psychiatry, Institute of Psychiatry and Mental Health, Hospital General Universitario Gregorio Marañón; 9 School of Medicine, Universidad Complutense, IiSGM, Madrid, Spain; 10 Department of Psychiatry, Melbourne Neuropsychiatry Centre, The University of Melbourne & Melbourne Health, Victoria, Australia; 11 Department of Psychiatry, Hospital Santiago Apostol, Vitoria; 12 Department of Psychiatry, Puerta de Hierro University Hospital-Majadahonda , ITA Mental Health, Madrid; 13 Instituto de Investigación, Transferencia e Innovación, Ciencias de la Salud y Escuela de Doctorado, Universidad Internacional de La Rioja, Logroño, Spain; 14 Institute of Psychiatric Phenomics and Genomics (IPPG), University Hospital, LMU Munich; 15 Department Clinical Translation, Max Planck Institute of Psychiatry, Munich, Germany

## Abstract

**Introduction:**

Childhood maltreatment (CM) is a widely recognized risk factor for mental disorders across the lifespan, although the underlying neurobiological mechanisms remain partially understood. The hypothalamic-pituitary-adrenal (HPA) axis is the primary physiological stress response system, and genetic and epigenetic variability in associated genes, such as *FKBP5* and *NR3C1,* have been proposed as mechanisms of risk or resilience in interaction with environmental exposures.

**Objectives:**

i) to investigate whether CM leaves detectable epigenetic marks in exposed children and adolescents, genome-wide and in candidate genes, and (ii) to examine how epigenetic variability in *FKBP5* and *NR3C1* modulate HPA-axis diurnal and psychosocial stress responses.

**Methods:**

203 children and adolescents were enrolled in the Epi_Young_Stress_Project (130 with psychiatric diagnoses and 81 controls). Cortisol diurnal profiles were assessed using four saliva samples collected throughout the day. Psychosocial stress responses were measured with five samples during the Trier-Social-Stress test. DNA methylation was quantified in PBMCs using Illumina EPIC_array_v1. Epigenome-wide association analyses (EWAS) were conducted using “Limma”. Principal component analyses were applied to characterize gene-level variability in candidate genes. Linear regression models were used for the study aims.

**Results:**

EWAS analyses revealed 29 differentially methylated positions between maltreated and non-maltreated youths, although none of them survived FDR correction. CM was associated with methylation in *FKBP5*, but not *NR3C1.* Diurnal cortisol profile was related to methylation in both genes, and interaction effects between genes were detected. TSST cortisol profile was associated with *FKBP5* methylation*.*

**Conclusions:**

CM leaves epigenetic signatures HPA-axis related genes, which may influence diurnal and psychosocial cortisol secretion in youths.

**Disclosure of Interest:**

None Declared

